# Outcomes of Sjögren’s versus non-Sjögren’s related dry eye in a longitudinal, tertiary clinic-based sample

**DOI:** 10.1371/journal.pone.0261241

**Published:** 2021-12-17

**Authors:** David Cui, Priya Mathews, Gavin Li, Shanna VanCourt, Esen Akpek

**Affiliations:** Ocular Surface Disease and Dry Eye Clinic, The Wilmer Eye Institute, The Johns Hopkins University School of Medicine, Baltimore, Maryland, United States of America; University of Toronto, CANADA

## Abstract

**Purpose:**

To assess the long-term treatment outcomes of dry eye in patients with and without underlying primary Sjögren’s syndrome (SS).

**Design:**

Retrospective longitudinal cohort.

**Methods:**

SS and non-SS dry eye patients with clinic visits for a minimum of 5 consecutive years at a tertiary, dedicated dry eye clinic were included. Electronic health records were reviewed to collect data regarding demographics, objective dry eye parameters, treatments utilized at baseline and final visit, and corneal complications observed during follow-up.

**Results:**

Two hundred and two patients (101 SS and 101 randomly selected non-SS), with a mean follow-up of 7.1 years were included. At baseline, mean conjunctival lissamine green staining score was 2.9 and mean corneal fluorescein staining score was 2.0. At last visit, notable improvement in staining score for cornea (–1.1, P < .001) and conjunctiva (–1.8, P < .001) was seen equally in both dry eye groups. Most patients (88.1%) had an escalation of treatment by the final visit, with similar rates in both groups (P = .51). Half (48.9%) of the patients had no conjunctival staining, and a third (34.4%) had no corneal staining at their last visit. Twenty (9.9%) patients experienced a vision-threatening corneal complication, including ulcers and melt, with no difference in occurrences between the groups (P = .64).

**Conclusions:**

The majority of patients in this longitudinal, tertiary clinic-based sample demonstrated improvement in their ocular surface staining score by the final visit with escalation in treatment. Treatments used, improvement achieved, and corneal complication rates leading to loss of vision were similar in both SS and non-SS dry eye groups.

## Introduction

Dry eye is a common ocular condition that has been associated with impaired daily function, physical and emotional distress, and decreased quality of life [1–6]. Epidemiologic studies have estimated that dry eye affects 5% to 50% of the global population and is a leading cause of visits to an eye care provider [7–9]. Although patients frequently complain of blurred or fluctuating vision, dry eye does not typically cause decreased visual acuity. Importantly, dry eye has a substantial financial burden on the U.S. healthcare system, with an estimated treatment and healthcare cost of $3.8 billion per year and total cost (including reduced productivity and work absence) at an estimated $55.4 billion per year [7, 10]. A single prescription dry eye treatment (Restasis^®^, Allergan Inc.) accounted for 29.5% of all ophthalmic medication expenditures in 2015 [11]. Many of the health care insurance policies do not provide coverage for dry eye treatments and the average monthly cost for individual patient ranges from $678 to $1267 based on disease severity [7, 10].

Despite the high cost to individual patients and society, the long-term efficacy of dry eye treatments remains insufficiently studied. There are no published prospective studies to our knowledge that assess the long-term outcomes of dry eye or treatments used. As there is no consensus regarding which objective parameters have the greatest validity to diagnose dry eye and monitor severity over time, it is difficult to determine whether dry eye is progressive over the long term or whether treatment might alter its natural course [2, 9, 12]. Furthermore, while prescription treatments for dry eye demonstrated improvement in short-term clinical trials to gain regulatory approval, studies regarding the long-term effectiveness for these costly prescription treatments are scarce and include limited data [2, 9, 12–17]. A single published study on the natural history of dry eye focused on participant-reported outcomes and found that approximately a quarter of subjects had worsening vision-related symptoms [15]. The study did not disclose changes in objective dry eye parameters or any treatments patients received [15]. Therefore, the long-term outcome of dry eye patients treated in a dedicated tertiary clinic warrants further study and serves as the primary objective of our paper.

As an additional study objective, we aimed to compare Sjögren’s syndrome (SS) dry eye to non-SS dry eye for severity and progression. Although the etiology of dry eye is diverse and includes natural causes such as aging, lifestyle factors such as contact lens wear or computer use, SS is a notable cause of aqueous-deficient dry eye secondary to an autoimmune etiology [7, 8, 18]. Sjögren’s syndrome is diagnosed in approximately 10% of patients with clinically significant dry eye, which is defined as presence of abnormal physician quantified dry eye parameters including Schirmer’s test, conjunctival or corneal vital dye staining, or abnormal tear film break-up time [19–22]. Although SS dry eye is generally regarded as more serious than non-SS dry eye [23, 24], it is not known whether long-term outcomes of SS dry eye are worse than non-SS dry eye. Existing literature is limited to the measurement of tear film inflammatory markers or cross-sectional clinical dry eye studies [3, 23, 24]. Information on how SS compares clinically with non-SS dry eye, including the longitudinal clinical course, is an additional area that needs investigation.

We herein aimed to summarize treatment patterns in SS versus non-SS dry eye disease and assess the long-term clinical outcomes at our tertiary care Ocular Surface Disease and Dry Eye Clinic at the Johns Hopkins Wilmer Eye Institute.

## Methods

The Johns Hopkins University Institutional Review Board (IRB) approved this retrospective chart review. The IRB waived the requirement for informed consent since the study was a retrospective chart review. The data was fully anonymized prior to analysis. The study was conducted in adherence with the Declaration of Helsinki and the Health Insurance Portability and Accountability Act.

Patients with a primary diagnosis of dry eye and long-term clinical follow-up, defined as having a minimum of one visit per calendar year for five consecutive years at the Ocular Surface Disease and Dry Eye Clinic at the Johns Hopkins Wilmer Eye Institute, were included. Patients with dry eye secondary to other ocular surface diseases (e.g., graft-versus-host disease, mucous membrane pemphigoid, Stevens-Johnson syndrome) as well as patients with associated systemic inflammatory/autoimmune diseases other than SS (e.g., rheumatoid arthritis, systemic lupus erythematosus, scleroderma, sarcoidosis) were excluded. All SS patients had to have a definitive diagnosis of primary SS with complete testing. Objective dry eye parameters included Schirmer’s test without anesthesia, tear film osmolarity, conjunctival lissamine green staining, and corneal fluorescein staining, in the order listed here. Conjunctival and corneal staining scores were graded using the previously published Ocular Surface Staining system [25]. A combined ocular surface staining score (0 to 12) was calculated by summing the conjunctival (0 to 6) and corneal (0 to 6) staining components [25]. Conjunctival staining was obtained by summing nasal (0 to 3) and temporal (0 to 3) conjunctival staining [25]. Corneal staining was calculated by summing the punctate staining grade (0 to 3) with +1 grade added for each of the following: central staining, confluence staining, and filaments [25]. For the purpose of this study, only the right eye parameters were used for each patient.

Electronic medical records were reviewed in detail by two trained research staff members (D.C. and G.L.). Data collected included demographic information, SS status and dry eye history, objective dry eye parameters, dry eye treatments utilized at baseline versus final visit, and corneal complications during follow-up. For the purpose of this study, treatments were categorized by the dry eye treatment level severity used in clinic (Table 1), which was adapted from the Tear Film and Ocular Surface Society Dry Eye Workshop II consensus recommendations [26].

**Table 1:** Classification of treatment levels used according to severity of objective dry eye parameters in a longitudinal, clinic-based sample of patients with Sjögren’s and non-Sjögren’s dry eye disease.

Level 1	Modification of local environment and medication use, dietary modifications, over-the-counter eye drops and lubrication, lid hygiene and warm compress
Level 2	In addition to the above, preservative-free ocular lubricants, tea tree oil, punctal occlusion, moisture chamber spectacles, overnight ointments or moisture chamber devices, in-office procedures, and topical prescription drugs or oral antibiotics
Level 3	In addition to the above, autologous serum eye drops, therapeutic contact lens, and oral secretagogues
Level 4	In addition to the above, amniotic membrane grafts, surgical punctal occlusion, topical corticosteroids for longer duration, and other surgical approaches (tarsorrhaphy, salivary gland transplantation)

We aimed to include all eligible patients with SS dry eye in this study. Nine hundred and thirty patients were identified using an existing SS database created in 2008 that is maintained and regularly updated by the research team at the Jerome L. Green Sjögren’s Syndrome Center at the Johns Hopkins Bayview Medical Center. Sjögren’s syndrome status was based on the strict 2012 criteria by the American College of Rheumatology, which requires two of the following: 1) positive serology (Sjögren-specific antibody A, Sjögren-specific antibody B, or rheumatoid factor+anti-nuclear antibody [titer 1:320]), 2) positive labial salivary gland biopsy (focal lymphocytic sialadenitis with focus score ≥ 1 focus/4mm^2^, and 3) presence of significant dry eye (ocular staining score ≥ 4) [27]. One hundred and one SS dry eye patients with full dry eye evaluations and sufficient follow-up qualified to be included in the review.

Non-SS dry eye patients were identified from a clinical sample among patients seen at the Ocular Surface Disease and Dry Eye Clinic at the Johns Hopkins Wilmer Eye Institute using the billing codes for dry eye (H16.223, H04.123, 370.33, 375.15). A statistical software was used to randomly select 101 qualified patients to be included in the review.

Data analysis was performed using STATA software version 14 (STATA Corp, College Station, TX). One-tailed student’s *t* test (for normally distributed data) and Wilcoxon rank-sum test (for not normally distributed data) were used to compare all quantitative variables including tear osmolarity, Schirmer’s, and ocular surface staining. Pearson’s χ^2^ was used to evaluate categorical variables such as demographic and treatment information. All P values less than .05 were deemed statistically significant. Values were compared between the first and the final visit to assess for change over time, as well as between the two cohorts (SS vs non-SS dry eye) for both initial and final presentation.

## Results

One hundred and one primary SS- and an equal number of randomly selected non-SS dry eye patients were included. The mean follow-up period was 7.1 years (median 6.4; range 3.8–15.4 years). Table 2 shows the baseline demographic information of the two cohorts. The majority of the patients were women and self-identified as White, with the SS dry eye cohort having more women (95.0% vs 82.2%, P = .004). The mean age at the baseline visit was older for non-SS dry eye compared with SS dry eye patients (53.0 vs 59.7, P < .001). There was no difference with regard to race, ethnicity, or smoking status between the two cohorts. Two-thirds (66.3%) of SS patients had prior diagnosis or were specifically referred for evaluation of SS at presentation. The remaining one-third received their diagnosis upon presenting for dry eye evaluation or during follow-up.

**Table 2:** Demographic characteristics of a longitudinal, clinic-based sample of patients with Sjögren’s and non-Sjögren’s dry eye disease.

Demographic	All Dry Eye Patients (n = 202)	Sjögren’s Dry Eye (n = 101)	Non-Sjögren’s Dry Eye (n = 101)	P Value^a^
Women, n (%)	179 (88.6)	96 (95.0)	83 (82.2)	.004
Mean age at presentation ± SD	56.4±13.4	53.0±12.5	59.7±13.6	< .001
Race, n (%)^b^				-
White	162 (78.6)	79 (76.0)	83 (81.4)	-
Black	24 (11.7)	15 (14.4)	9 (8.8)	-
Indian/Alaskan Native	1 (0.5)	1 (1.0)	0	-
Asian	9 (4.4)	3 (2.9)	6 (5.9)	-
Other	10 (4.9)	6 (5.8)	4 (3.9)	-
Hispanic or Latino ethnicity, n (%)	17 (8.4)	8 (7.9)	9 (8.9)	-
Smoker, n (%)	6 (3.0)	4 (4.0)	2 (2.0)	-

SD = standard deviation.

^a^P values were >.10 unless otherwise indicated.

^b^Four participants self-identified with more than one race.

Table 3 demonstrates the objective dry eye parameters at baseline versus final visit for both cohorts. As a trend, although both cohorts had significant dry eye at baseline, SS dry eye patients consistently had worse parameters (e.g., lower Schirmer’s results, higher ocular surface staining, higher tear osmolarity) compared with the non-SS cohort. Compared with baseline, patients in both cohorts had improvement in objective dry eye parameters at their final visit. The improvement was statistically significant for both corneal and conjunctival staining scores in both cohorts (P < .001). A total of three patients (1.5%) had worsening of ocular surface staining as of last visit.

**Table 3:** Baseline and final objective dry eye parameters for a longitudinal, clinic-based sample of patients with Sjögren’s and non-Sjögren’s dry eye disease.

Ocular Surface and Tear Film Parameter	All Patients (n = 202)	Sjögren’s Dry Eye (n = 101)	Non-Sjögren’s Dry Eye (n = 101)	P Value^a^
Baseline osmolarity	305.1±14.4	306.1±15.8	304.4±13.2	.06
Final osmolarity	303.2 ±17.6	306.5±12.2	301.4±19.6	.05
Change in osmolarity^b^	–3.0±19.4	+.5±17.9	–4.2±20.2	-
Baseline Schirmer’s	9.6±9.0	8.2±8.9	11.9±8.8	.006
Final Schirmer’s	11.8±9.9	10.6±9.5	13.6±10.7	-
Change in Schirmer’s^b^	+1.9±14.1	+1.7±15.4	+2.5±11.7	-
Baseline conjunctival staining	2.9±2.2	3.5±2.2	2.4±2.0	.001
Final conjunctival staining	1.1±1.6	1.6±1.9	.6±1.2	< .001
Change in conjunctival staining ^b^	–1.8±2.1	–2.0±2.2	–1.7±2.1	-
	P < .001	P < .001	P < .001	
Baseline corneal staining	2.0±1.4	2.2±1.4	1.9±1.3	-
Final corneal staining	.9±1.1	1.0±1.1	.7±1.0	.05
Change in corneal staining^g^	–1.1±1.6	–1.1±1.7	–1.2±1.6	-
	P < .001	P < .001	P < .001	
Baseline combined ocular surface staining	4.5±3.1	5.0±3.4	4.1±2.8	.10
Final combined ocular surface staining	1.8±2.2	2.4±2.6	1.3±1.6	.002
Change in combined ocular surface staining	–2.7±3.3	–2.7±3.6	–2.8±3.0	-
	P < .001	P < .001	P < .001	

Values recorded in mean±standard deviation.

^a^P values were >.10 unless otherwise indicated.

^b^Changes in objective dry eye parameters were >.10 unless otherwise indicated.

By the final visit, half (48.9%) of all patients had no conjunctival staining, a third (34.4%) had no corneal staining, and a quarter (26.7%) had no ocular surface staining. More non-SS than SS patients had resolved conjunctival (65.6% vs 31.0%, P < .001) and combined ocular surface (34.4% vs 18.4%, P = .02) staining.

Table 4 depicts the prevalence and frequency of each dry eye treatment at baseline and final visit, and procedure/device use during or before visit. Compared to baseline visit, more patients at the final visit were on topical anti-inflammatory/immunomodulating agents, autologous serum tears, in-office procedures or at-home device use, and surgical procedures (all P < .05). The only decrease in treatment prevalence was with the number of patients using over-the-counter tears, gels, and ointments, which still was the most commonly used treatment at both time points (75.3% final visit vs 90.6% baseline visit, P < .001). Topical cyclosporine 0.05% (21.3% baseline vs 57.9% final visit, P < .001) and punctal plugs (11.4% baseline vs 55.9% final visit, P < .001) were the most commonly used prescription treatment and in-office procedure respectively. All patients on oral secretagogues, at baseline and final visit, were in the SS cohort.

**Table 4:** Dry eye treatment prevalence and dosage frequency at baseline versus final visit for a longitudinal, clinic-based sample of patients with Sjögren’s and non-Sjögren’s dry eye disease.

Treatment	Baseline Visit		Final Visit		Change in Prevalence
	Prevalence	Mean Daily Dosage	Prevalence	Mean Daily Dosage	
	n (%)	n	n (%)	n	P Value^a^
Over-the-counter tears, gels, and ointments	183 (90.6)	3.5	152 (75.3)	3.8	< .001
Topical anti-inflammatory/immunomodulatory treatments^b^	44 (21.8)	2.6	135 (66.8)	2.7	< .001
Topical corticosteroids^c^	15 (7.4)	2.3	16 (7.9)	2.2	-
Topical anti-histamine/mast cell stabilizers^d^	6 (3.0)	1.6	13 (6.4)	1.7	-
Topical antibiotics^e^	9 (4.5)	1.2	13 (6.4)	1.9	-
Oral antibiotics	0	-	3 (1.5)	-	-
Oral secretagogues	23 (11.4)	-	25 (12.4)	-	-
Autologous serum tears	4 (2.0)	2.7	18 (8.9)	3.3	.002
In-office procedures^f^	24 (11.9)		129 (63.9)		< .001
Home-based device use^g^	2 (1.0)	-	17 (8.4)	-	< .001
Surgical procedures^h^	3 (1.5)	-	31 (15.3)		< .001
Systemic anti-inflammatory treatments (Sjögren’s cohort only, n = 101)^i^	36 (35.6)	265 mg	57 (56.4)	299 mg	.003

^a^P values were >.10 unless otherwise indicated.

^b^Cyclosporine .05%, cyclosporine .09%, cyclosporine 1% or 2%, lifitegrast, tacrolimus .01.

^c^Loteprednol, difluprednate, fluorometholone, prednisolone.

^d^Olopatadine, ketotifen, azelastine.

^e^Erythromycin, azithromycin, bacitracin.

^f^Punctal plugs, Blephex^®^, Lipiflow^®^, Intense Pulsed Light.

^g^Scleral lens, True Tear^®^.

^h^Tear duct cauterization, tarsorrhaphy.

^i^Hydroxychloroquine, methotrexate, cyclophosphamide, rituximab.

**Fig 1:**
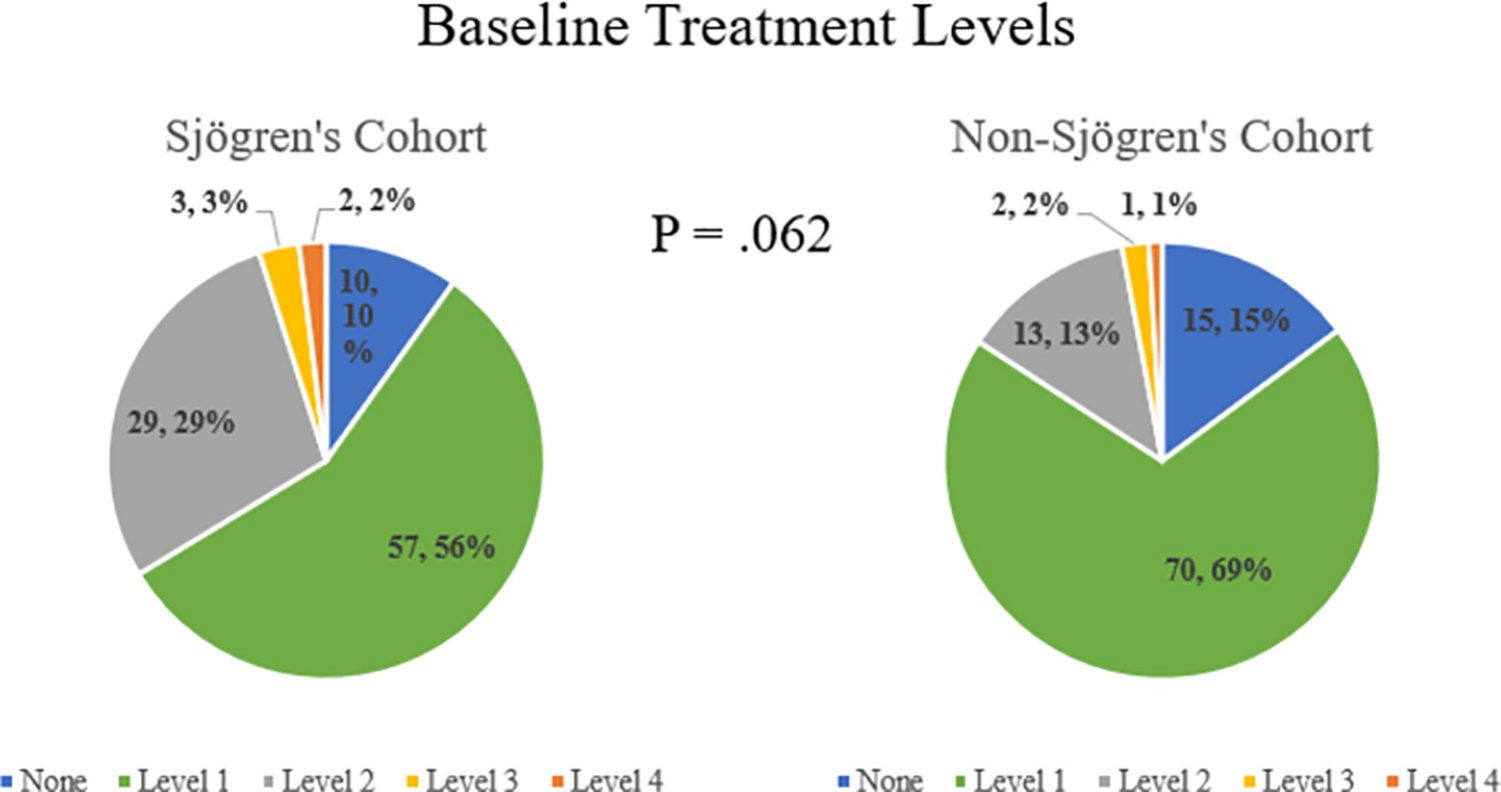
Baseline treatment levels according to tear film & ocular surface society treatment level classification for a longitudinal, tertiary clinic-based sample of patients with Sjogren’s and non-Sjogren’s dry eye disease (n = 202).

**Fig 2:**
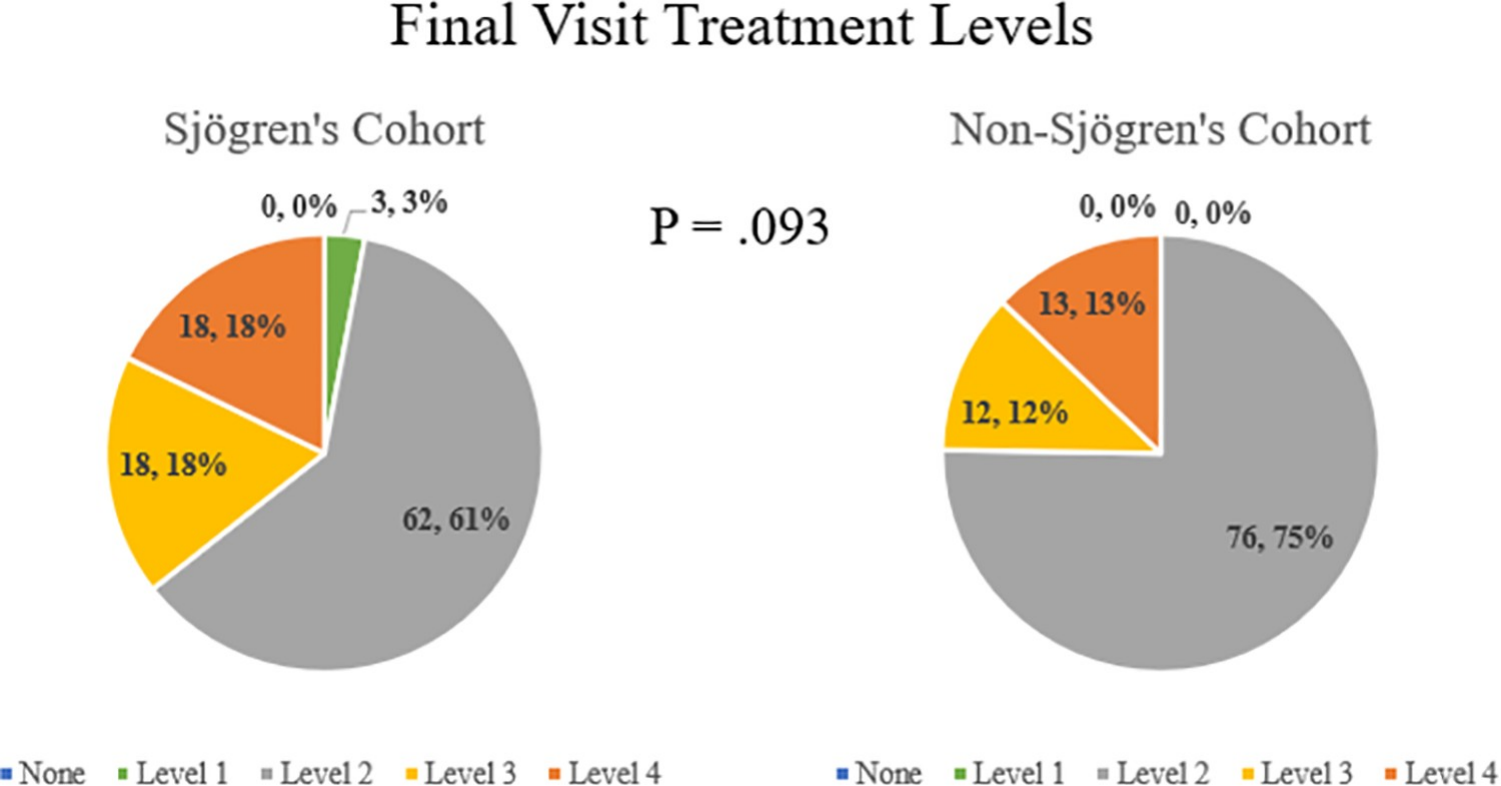
Final treatment levels according to tear film & ocular surface society treatment level classification for a longitudinal, tertiary clinic-based sample of patients with Sjogren’s and non-Sjogren’s dry eye disease (n = 202).

Treatments utilized according to dry eye severity level are depicted in Fig 1 for the baseline visit and Fig 2 for the final visit. Secretagogues were only prescribed for SS patients in this cohort, often for systemic dryness and xerostomia and not solely for dry eye. They were not included in determining TFOS treatment level in this study, as the treatment levels were designed for evaluating general dry eye. At both the baseline and final visit, there was no difference between the SS and non-SS cohorts with regard to treatment level (P = .06 and .09, respectively). A large majority of all patients (88.1%) were on an elevated treatment level by final visit compared to baseline visit, with no difference between the two cohorts (90.1% SS vs 86.1% non-SS, P = .51).

During follow-up, 20 (9.9%) patients experienced 25 episodes of vision-threatening corneal complications, which consisted of 12 episodes of corneal epithelial defects/ulceration and 2 corneal melt/perforation. Eleven patients lost vision due to corneal haze/scarring on follow-up. Interestingly, approximately half of patients with corneal complications had non-SS dry eye (11 non-SS vs 9 SS, P = .64). When comparing patients with corneal complications to those without, there was no difference with regard to sex, treatment level escalation, or objective dry eye parameters at baseline or final visit (all P > .05).

## Discussion

This longitudinal, tertiary clinic-based retrospective study suggests that with proper escalation of dry eye treatment, both SS and non-SS dry eye patients achieve significant improvement of objective dry eye parameters. Nearly half (48.9%) of patients had resolved conjunctival staining, a third (34.4%) had resolved corneal staining, and a quarter (26.7%) had no ocular surface staining by their final visit. However, despite vigilant management strategies, 20 patients experienced 25 episodes of corneal complications, and 11 had permanent decrease of vision due to scarring/haze, with no difference between SS versus non-SS dry eye cohorts.

Long-term effectiveness of currently available dry eye treatments is unknown, despite the significant costs to the patients and the healthcare system. This is partly due to the lack of a validated or uniformly agreed upon objective dry eye parameters to assess dry eye severity over time [2, 7, 9, 15, 28]. As a result, it is difficult to define and assess dry eye worsening and to study the efficacy of treatments and procedures compared to no treatment or simple over-the-counter medications. Current literature explores the progression of dry eye from a patient perspective, with one study reporting that approximately a quarter of participants have progressive dry eye defined by worsening of ocular discomfort or visual symptoms [15]. A second study, Progression of Ocular Findings, is an observational study that evaluated dry eye progression based on objective dry eye parameters and patient reported symptoms [9]. Participants in the study did not receive clinical care for dry eye and therefore there was no assessment of treatment outcomes [9]. Only the baseline findings of this study have been published thus far [9].

Our results indicate that objective dry eye parameters improve over time as a trend with appropriate management strategies. However, although the changes were statistically significant for ocular surface staining scores, the clinical significance remains unknown. In addition, the lack of correlation between patient-reported symptoms and physician-measured dry eye parameters makes it difficult to assess patient satisfaction [29–31].

By the final visit, the treatment was escalated in most (89.1%) of the patients. Almost all (98.5%) patients were on a Level 2 or higher treatment regimen at final visit, which typically includes a prescription treatment or an in-office procedure. These treatments regimens may be costly or require visits to an eye care provider. The utilization of multiple concurrent treatments makes it difficult to isolate the treatment with the greatest efficacy and can lead to increased cost, clinic time, and frustration for both physician and patient. Furthermore, these patients may be required to stay on their treatment regimens for a long or unknown period of time. Therefore, long-term prospective clinical trials comparing various treatment modalities is necessary.

Our study supports existing literature that SS is associated with worse clinical dry eye parameters compared to non-SS dry eye [23, 24]. In this cohort SS patients were younger, perhaps indicating that dry eye starts at a younger age [7, 8]. However, we did not find a difference in the rate of vision-threatening corneal complication between the two cohorts (approximately 10% in both), suggesting that non-SS dry eye can also be quite severe.

Our results must be interpreted with caution due to the several limitations of the study design. This study is a retrospective review of health care records. The patients included were evaluated at a specialty dry clinic at a tertiary referral center and may have more severe clinical findings. Treatment methods utilized are in line with the TFOS DEWS II recommendations [26]. Oral secretagogues were not included in the analyses as only SS patients were on these medications. Treatment regimens used may not be offered by all eye care providers, due to issues with availability or patient access. Non-SS dry eye patients were randomly selected and not matched, although matching by age would have selected for younger non-SS patients, creating a greater difference in observed objective dry eye parameters. Further, our inclusion criteria selected patients who had adequate and consistent clinical follow-up. In doing so, we may have selected patients with more symptomatic or worse ocular health as patients with resolved or mild symptoms may not have had regular follow-ups. Likewise, patients who had chronic dry eye refractory to our treatment recommendations may have discontinued visits or sought care at another institution due to dissatisfaction. In addition, we cannot definitively state that those in the non-SS cohort do not have any autoimmune disease, as not all in the cohort received full work-up to definitively rule out systemic disease. We did not analyze results of each specific treatment, instead classified the treatments according to the DEWS II designation which makes it difficult to draw conclusions due to heterogeneity. Finally, we did not collect information systematically on the patients’ symptoms. Because patient-reported symptoms do not always correlate with objective dry eye parameters [29–31], we cannot comment on whether or not patients felt that they had improvement with the treatments offered. Nevertheless, this is a large, longitudinal cohort of dry eye patients with consistent follow-up who received comprehensive dry eye evaluations and somewhat standardized treatment according to a previously published algorithm [26].

In conclusion, the severity of dry eye, as measured by ocular surface staining, can improve over time with adequate treatment and close regular follow-up. Treatment requirements and outcomes including vision threatening corneal complication rates seem similar between the SS and non-SS dry eye cohorts.
